# Cognitive, behavioral and psychiatric symptoms in patients with spinal cord injury: a scoping review

**DOI:** 10.3389/fpsyt.2024.1369714

**Published:** 2024-03-20

**Authors:** Andrea Calderone, Davide Cardile, Rosaria De Luca, Angelo Quartarone, Francesco Corallo, Rocco Salvatore Calabrò

**Affiliations:** ^1^ Department of Clinical and Experimental Medicine, University of Messina, Messina, Italy; ^2^ IRCCS Centro Neurolesi Bonino-Pulejo, Messina, Italy

**Keywords:** spinal cord injury, cognitive symptoms, psychiatric symptoms, neurorehabilitation, mental health

## Abstract

Spinal Cord Injury (SCI) is a condition where the spinal cord is damaged and experiences partial or complete loss of motor and/or sensory function, which is typically less than normal. After SCI, patients may exhibit more severe psychiatric symptoms and experience cognitive impairments, including reduced speed and attention processing capacity, as well as difficulties with executive function and episodic memory retention. Among the behavioral and psychiatric symptoms, depression, anxiety, substance use disorder, and posttraumatic stress disorder are the most common. This review aims to investigate the cognitive, behavioral, or psychiatric symptoms of the patient with SCI and their influence on the rehabilitation process. Studies were identified from an online search of PubMed, Web of Science, Cochrane Library, and Embase databases. Studies published between 2013-2023 were selected. This review has been registered on OSF (n) 3KB2U. We have found that patients with SCI are at high risk of cognitive impairment and experience a wide range of difficulties, including tasks based on processing speed and executive function. This clinical population may experience adjustment disorders with depression and anxiety, as well as other psychiatric symptoms such as fatigue, stress, and suicidal ideation. This review has demonstrated that SCI patients may experience psychiatric symptoms and cognitive impairments that affect their functioning. At the same time, these patients may be more prone to various adjustment and mood disorders. Moreover, these two aspects may interact with each other, causing a range of symptoms, increasing the risk of hospitalization, and delaying the rehabilitation process.

## 1 Introduction

Spinal cord injury (SCI) is a devastating neurological condition that causes physical dependence, morbidity, and psychological stress and occurs when the spinal cord is damaged, usually below the level of injury, leading to partial or complete loss of motor and/or sensory function (1). Over the last 30 years, the global prevalence has increased from 236 to 1,298 cases per million population, whereas the global incidence of SCI is estimated to have increased from 250,000 to 500,000 cases per year (2). Before the 1940s, only 10% to 20% of people survived more than a few weeks after injury (3). Technological advances have significantly improved this condition, with 90% of people now surviving for more than a year after injury and around 50% surviving for 40 years after injury (4). SCI has a severe impact on physical well-being, leading to decreased physical functioning and increased pain (5, 6). Maladaptive emotional and/or behavioral responses to identifiable psychosocial stressors, such as SCI or other stress-related events, are hallmarks of adjustment disorder, which results in maladjustment following an event that is disproportionate to the stressor. They are characterized by stress responses that deviate from socially or culturally expected responses to the stressor and/or cause significant distress and impairment in daily functioning (7). Mood is defined as a broad and persistent emotional tone that persists internally and affects almost all aspects of a person’s behavior in the external world. Mood disorders are expressed by significant emotional disturbances (severe lows, called depression, or highs, called hypomania or mania). These include bipolar disorder, cyclical hypomania, hypomania, major depressive disorder, mood dysphoria, persistent depressive disorder, and premenstrual dysphoric mood disorder. These are common mental disorders leading to increased morbidity and mortality, and mood disorders are broadly divided into bipolar and depressive disorders (8).

These disorders may be present in SCI patients who experience increased depression, stress, and anxiety following an injury (9, 10). Resulting psychiatric symptoms are common in this clinical population (11–13) reflecting significant changes in general well-being after injury (14). Furthermore, up to 50% of patients with SCI appear to have cognitive impairment (15–18). Common cognitive problems include decreased processing speed and attention, and difficulties in episodic memory and executive function (19–27). According to a recent report, the risk of cognitive impairment in patients with SCI is approximately 13 times higher than in healthy individuals. Moreover, cognitive impairment is a strong predictor of inadequate social participation after hospital discharge, with an 8.4:1 probability of being unable to work (28). Impairments in attention, concentration, memory, problem-solving, and reasoning tend to be most common, but there are significant individual differences in cognitive functioning after SCI and a wide range in performance depending on the severity and pattern of affected areas (29). Traumatic brain injury (TBI) comorbidity is often cited as a cause of cognitive impairment in people with SCI, as external forces causing trauma to the spinal cord (30, 31) sometimes result in TBI. However, research also shows that a significant number of people with traumatic or non-traumatic SCI and no history of TBI have neuropsychological dysfunction (32, 33). Studies in human and animal models suggest that cognitive impairment in patients with SCI may be a consequence of pathophysiological factors such as cortical remodeling and atrophy, neuroinflammation, hypoxia, vascular dysfunction, and accelerated aging (34–36). Furthermore, treatment-related factors (including medications prescribed during acute and chronic treatment), sequelae of SCI (e.g., sleep disorders and chronic pain), and pre-onset conditions (e.g., psychiatric disorders and learning difficulties) may contribute to the development of cognitive impairment after SCI (37). Patients with moderate to severe cognitive impairment have been reported to have difficulty learning skills during rehabilitation, experience more disturbances in sleep and appetite, require higher levels of care, and have reduced functional independence (38, 39). One tool that can be used to create a baseline cognitive profile for people with SCI is the Repeatable Battery for Assessment of Neuropsychological Status (RBANS), which is not suitable for the cognitive assessment of people with SCI with upper motor dysfunction, as it includes several subtests of this domain (40, 41). Primary and Secondary Disability Rating Scales (ADAPSS) are also available to cover important stressors specific to the SCI population (42). To detect anxiety and depression, the Hospital Anxiety and Depression Scale (HADS) can also be used (43). A summary of these three tools is displayed in **Supplementary Table 1**.

**Figure 1:**
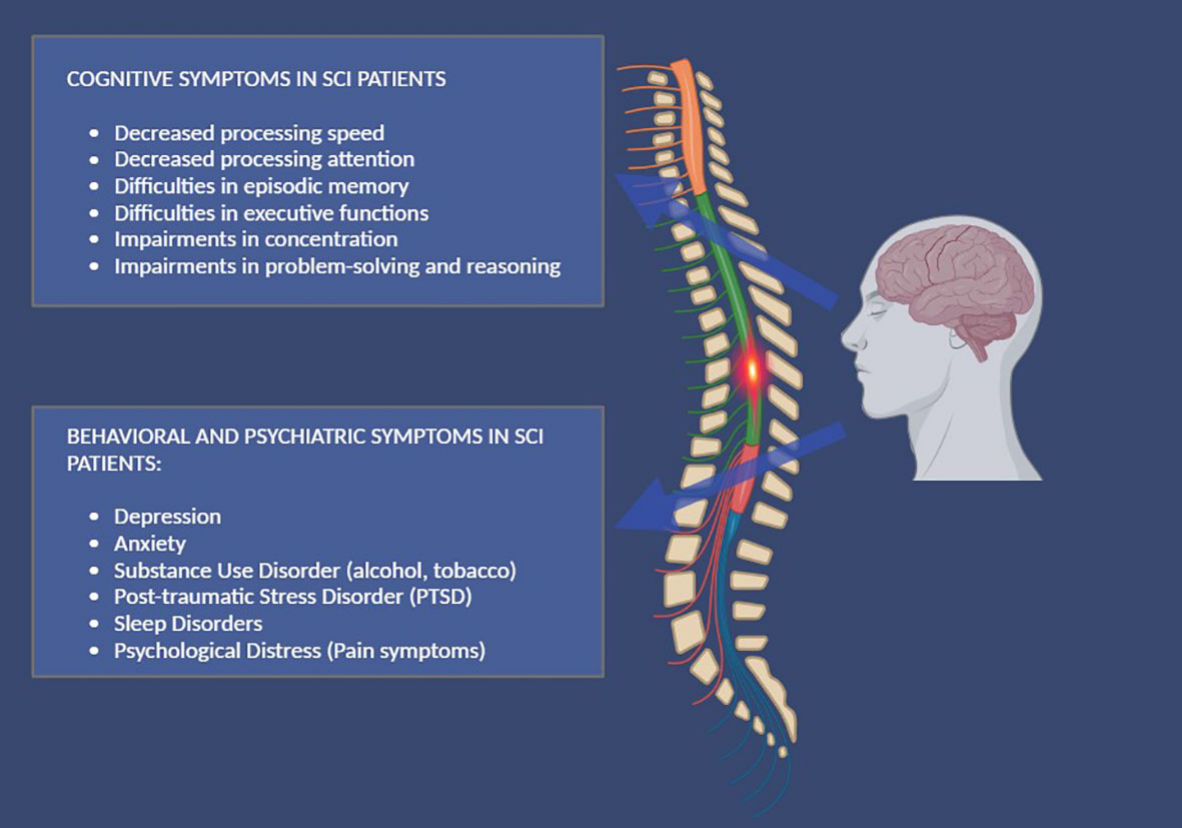
A summary of cognitive, behavioral, and psychiatric symptoms, in patients with SCI.

Depression is a common secondary complication after SCI (44) and is associated with poorer health status (45), reduced functioning (46, 47), and increased mortality (48). SCI patients with probable major depression are known to be more likely to report a history of other psychiatric disorders than those without probable major depression. Depression is often associated with diagnoses of other psychiatric disorders such as anxiety disorders and substance use disorders (49). Anxiety is a problem for adults with acquired SCI, with 45% of injured individuals reporting experiencing excessive worry, fear, or panic (50) and a high risk of experiencing disorders such as generalized anxiety disorder. The traumatic nature of SCI ongoing fear of life-threatening secondary outcomes, or pre-injury psychological morbidity can cause increased distress (51–53). Instead, post-injury psychological distress can be so intense and unbearable that patients may seek relief through the consumption of substances such as alcohol, tobacco, and cannabis (54, 55). For alcohol, consumption patterns are similar to the general population in terms of gender and age, but the rate of “risky consumption” is higher in the SCI population (56, 57). Tobacco is the second most consumed substance by this clinical population (58). Its prevalence ranges between 19-40% of injured patients. Tobacco use, in this population is often associated with harmful alcohol consumption. Logically, this harms an individual’s health and increases the likelihood of suffering from other medical complications (59–61). Another psychiatric condition that may develop in this population is post-traumatic stress disorder (PTSD) (62). Research has found that quadriplegia is associated with a reduced risk of PTSD, while paraplegia is associated with an increased risk (63) Moreover, combat veterans are more likely to suffer from PTSD and experience more severe symptoms than non-combat zone veterans (64). A summary of cognitive and adjustment disorders in patients with SCI is shown in **Figure 1**.

All these symptoms and dysfunctional behaviors must be treated. Specialized medical care is essential in the early management of SCI to ensure survival and prevent unnecessary complications. However, once the acute phase has passed, medicine cannot provide a cure, and everyone must learn to live with the disability that presents itself in their environment and work on their cognitive limitations or dysfunctional behaviors and emotions.

This scoping review aims to update what is known in this field about the cognitive, behavioral, or psychiatric symptoms in patients with SCI and their influence on the rehabilitation process.

## 2 Materials and methods

### 2.1 Search strategy

**Figure 2:**
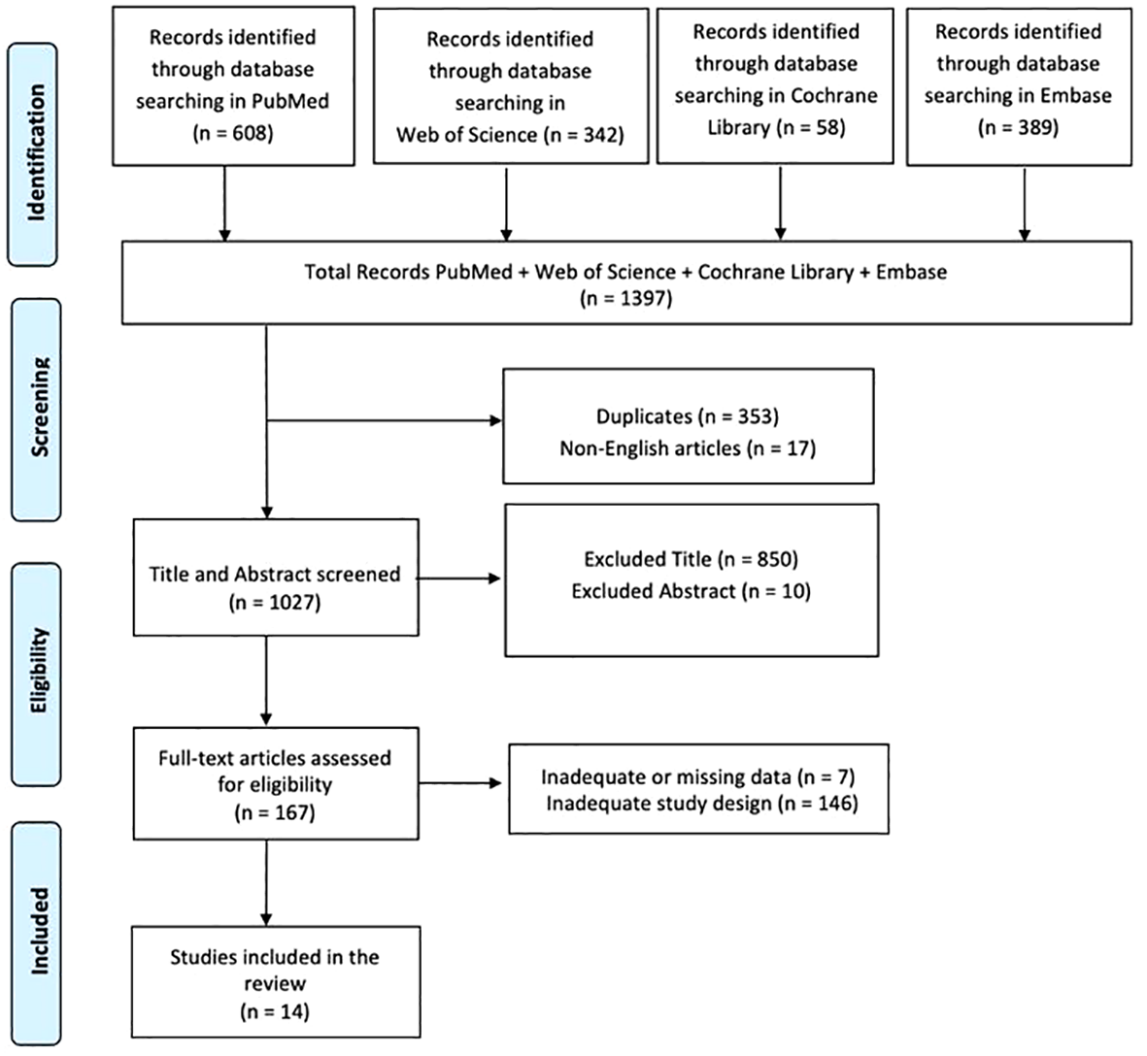
PRISMA 2020 flow diagram of evaluated studies.

A literature search was conducted via PubMed, Web of Science, Cochrane Library, and Embase, and it was carried out for articles using the following search keyword terms: (All Fields: “Spinal Cord Injury”) AND (All Fields: “Cognitive Symptoms”); (All Fields: “Spinal Cord Injury”) AND (All Fields: “Psychiatric Symptoms”) with 2013-2023 search time range. We adopted the PRISMA (Preferred Reporting Items for Systematic Reviews and Meta-Analyses) flow diagram to describe the sequence of steps (identification, screening, eligibility, and inclusion) for the collection and determination of qualified studies as shown in **Figure 2**. Titles and abstracts were independently scanned and retrieved from database searches. The suitability of the article was then assessed according to the defined inclusion criteria. Ultimately, we received all titles and abstracts that met the criteria for inclusion in the full text. To avoid bias, several expert teams worked together, selected the articles, and analyzed the data independently, and discussed any discrepancies with each other. Disagreements between reviewers were resolved by consensus.

This review has been registered on OSF (n) 3KB2U.

### 2.2 PICO evaluation

We defined our combination of search terms using a PICO (population, intervention, comparison, outcome) model. The population was limited to patients with moderate to severe SCI; the intervention included all studies, rehabilitation approaches, and assessment tools to measure and understand cognitive, behavioral, or psychiatric symptoms, and their influence on the rehabilitation process; the comparison was evaluated considering the different cognitive, behavioral and psychiatric symptoms in patients with SCI both before and during a psychological and motor rehabilitation process; and the result included any improvements in the identification of cognitive, behavioral and psychiatric symptoms as well as their evaluation, resolution or attenuation during the rehabilitation process.

### 2.3 Inclusion criteria

A study was included if it described or investigated the cognitive, behavioral, or psychiatric symptoms of a patient with SCI and their influence on the rehabilitation process. The review included only articles written in English. Studies describing or investigating the functional assessment of these patients were also included. We only included studies conducted in human populations that met the following criteria: (i) original or protocol studies of any type and (ii) articles that presented some cognitive, behavioral, or psychiatric symptoms of the patient with SCI and their influence on the rehabilitation process.

### 2.4 Exclusion criteria

A study was excluded if there was a lack of data or information about the description of the cognitive, behavioral, or psychiatric symptoms of the patient with SCI and their influence on the rehabilitation process. Systematic, integrated, or narrative reviews were also excluded, but reference lists were reviewed and included as necessary. Articles with cognitive and psychiatric symptoms purely due to TBI or other existing neurological conditions, such as Dementia, PD, or MS were also excluded. All articles written in languages other than English were finally excluded.

## 3 Results

In total, 1397 articles were found: 353 articles were removed due to duplication after screening; 17 articles were excluded because they were not published in English; 860 articles were excluded based on title and abstract screening. Finally, 153 articles were removed based on screening for inadequate study designs and untraceable articles (**Figure 2**). Fourteen research articles met the inclusion criteria and were therefore included in the review. A survey of these studies is shown in **Supplementary Table 2**.

The articles described in this review investigated the cognitive, behavioral, or psychiatric symptoms of the patient with SCI and their influence on the rehabilitation process. The cognitive symptoms of patients with SCI were analyzed in four articles (12, 41, 65, 66). The behavioral and psychiatric symptoms of these clinical populations are described in ten articles (67–76).

### 3.1 Cognitive symptoms in patients with SCI

Functional diagnosis of SCI patients involves the identification of cognitive symptoms that are relevant to their psychological functional status during rehabilitation. According to one study, these patients who resided in the community were found to be at a greater risk of mild cognitive impairment and faced certain challenges, including issues with processing speed, executive function-based tasks, and episodic memory tests. Furthermore, individuals with quadriplegia exhibit lower scores on cognitive tests of processing speed and executive function than those without paraplegic impairment (12). The second research discovered variations in cognition, age, and quality of life (QoL) between individuals with and without SCI. Those participants who did not have SCI had more favorable associations with QoL and cognition, while those with SCI did less poorly. Positive emotions/well-being and resilience were observed in those with SCI, along with a higher QoL. However, there was no difference in cognitive functioning between SCI and non-injured participants. This implies that individuals with SCI can adjust their QoL by decreasing the significance placed on mobility and cognitive impairment (65).The psychological effect of cognitive decline during acute rehabilitation is still evident in a prospective observational study of over 89 patients, which can have implications for the mental health of those with SCI. Through the use of RBANS and profile analysis, psychometric results indicated three groups that could be distinguished from each other by cognitive function. The majority of individuals (class 1 [54%]) did not experience any cognitive impairment in any domain. On the other hand, investigations revealed a population with late-onset memory impairment (class 2 [26%]) and cognitive impairment in different areas (Class 3 [20%]). Education, smoking, and drug use were strongly linked to cognitive impairment and classroom conditions. Those who had not been educated in high school had a history of drug use, smoked, and experienced more post-concussion symptoms were at heightened risk for enrollment in class 3 than in the first year of the program (41). A further study revealed that there was no overall correlation between neuropsychological test performance and symptom measures. Despite this, self-cognition was affected by anxiety and tiredness. Individuals who had cognitive impairment in one or more domains were found to have worse cognitive functioning when anxious. Those with cognitive impairment in at least one cognitive area reported lower working memory scores due to anxiety, while those with fatigue reported impaired delayed memory performance. Poor performance was also correlated with delayed recall, and cognitive performance showed no correlation with other depressive or fatigue symptoms (66).These first results demonstrate that the environment, together with age and education, in which people with this pathology live can negatively compromise cognitive functioning, increasing the probability of deterioration or further risks; however, they can implement processes of adaptation in their QoL by decreasing the importance or meaning attributed to their cognitive deficit. Levels of anxiety and fatigue can also influence cognitive performance so attention to these two aspects is essential to prevent or reduce other possible risk factors.

### 3.2 Behavioral and psychiatric symptoms in patients with SCI

Patients with SCI conditions may experience behavioral and psychiatric symptoms, which can lead to other disorders and clinical pathologies. In one study, was found that depression scores decreased 3 years after injury. Compared to the 1-year post-injury period, depression scores decreased in the 3 years after injury (T2) and 1 year (T1). This result suggests that the risk of depression in SCI patients increases with age, and the balance between cognitive and somatic symptoms of illness may be influenced by high autonomic fluctuations (67). A cross-sectional study found that individuals with SCI exhibit symptoms of depression and anxiety, which indicate lower levels of independence in exercise, personal hygiene, bowel control, and social interactions. A negative correlation was discovered with the Language subtest of the Montreal Cognitive Assessment Scale (MoCA) while the degree, nature, and duration of the injury were not correlated with alcohol or illicit drug use. The primary predictor of depression was the presence of anxiety, and SCI-related factors were not significant. Finally, characteristics of the Functional Independence Measure (FIM) and cognitive aspects of the MoCA scale were found to be the main predictors of depressive symptoms (68).A significant correlation was found between lower depressive symptoms and greater general self-efficacy and meaning in life. Through appraisal and coping strategies, it was observed that there were significant direct effects on higher life satisfaction and significant negative effects in individuals with depressive symptoms.The effect of general self-efficacy on depressive symptoms was fully mediated by appraisal and coping strategies (69). A study conducted in 2007 revealed that out of the 41,213 veterans surveyed, 2,615 had been diagnosed with SCI and depression, while 70% were also found to have other mental illnesses, with trauma Post-stress disorder and other anxiety disorders being the most prevalent. Veterans who suffered from SCI and depression were prone to frequenting more medical facilities and receiving additional medication than those who did not suffer from any depression (70). Another article highlights the connection between daily fatigue in these clinical populations, which results in heightened depressive symptoms reduced cognitive function, and a significantly lower level of social interaction during the event. Despite being socially engaged, anxiety and pain did not change significantly on that day. In comparison, when taking into account all symptoms, there was no correlation between daily changes in pain intensity or anxiety and social interaction on the same day. By altering the model to reflect age, gender, education level, injury category number, and time since the injury occurred, these effects were discovered (71). A multicenter study has revealed medically and psychologically related outcomes to pain. The presence of depression and anxiety symptoms at high levels is a reliable predictor of various pain factors, including gender, injury-related traits, and secondary comorbidities. Most patients with SCI endorsed lower levels of depression and anxiety on average. Moderate to severe depression was seen in 9.1% (suicidal ideation in 3.2%). Moderate to severe anxiety was seen in 8.0%. In addition, 28.3% were currently or in the past treated for or diagnosed with depression (5.9% for suicidal ideation and 3.2% for suicide attempts), while anxiety rates were slightly lower (22.5%). Only a small proportion of the sample (9.6%) reported receiving psychological treatment for mental health problems in the past year (72). A longitudinal study was conducted on 21 community-dwelling adults with chronic spinal cord injury at ages T1 (2004) and T2 (2009). Most were married, men, and completely paralyzed. Although some participants at T2 reported clinically significant psychiatric symptoms, none of the remaining participants at T1 met the eligibility criteria for T2. A 30-year-old woman with complete paralysis and non-traumatic SCI was found to be extremely stressed and depressed (18 years later), while a 64-year-old man suffered from a traumatic stress disorder and paralyzing limb weakness and was severely depressed (7 years after the injury) (73). In another article, 33% (n=21) of a sample of 63 SCI patients had suicidal ideation in the last two weeks; 71.4% (n=15) of suicidal ideation patients had depression; 52.4% (n=11) of patients with suicidal ideation were diagnosed with full-blown PTSD and 52.4% (n=11) of suicidal ideation patients had depression. Resilience was significantly lower in patients with depression and SI. While depression predicts suicidal ideation in traumatic SCI, resilience is a protective factor against SI (74). In a cross-sectional survey study, it was demonstrated that pain and fatigue were independently associated with depression, but only pain was associated with physical functioning. Furthermore, depression was more severe in middle-aged participants than in younger or older participants. Physical functioning decreased with increasing age and severity of injury (75). In one last comparative study of 37 patients with SCI, 89.2% (n. 33) of the patients had pain and 27.0% (n. 10) reported very severe pain; 9% had a history of psychiatric treatment for insomnia, depression, or anxiety after SCI but were currently receiving psychiatric treatment. The results found were that resilience can reduce the negative effects of pain. In addition, resilience independently contributed to reduced depression and posttraumatic growth (76). These results indicate that among the psychiatric symptoms that these patients may manifest are depression and anxiety. Regarding the first psychiatric pathology, we can state that its symptoms increase with age leading to autonomic fluctuations between cognitive/somatic deficits and lower levels in various aspects of functional and daily independence. However, the use of functional assessment and coping strategies leads to greater satisfaction. Patients with SCI are also more vulnerable to anxiety, daily fatigue, and pain, leading to a greater likelihood of developing depressive symptoms, including suicidal ideation, with high levels of stress, even years later. A greater focus on identifying and measuring the intensity and degree of these psychiatric symptoms becomes essential to prevent worse consequences.

## 4 Discussion

Our scoping review aimed to update what is known in this field about the cognitive, behavioral, or psychiatric symptoms in patients with SCI and their influence on the rehabilitation process. The studies included in this review have demonstrated that patients with SCI are at high risk of cognitive impairment and experience a wide range of difficulties, including tasks based on processing speed and executive function. Despite these difficulties, however, they appear to be able to readjust their QoL through positive affect and flexibility, minimizing the importance of mobility and cognitive impairment (12, 65). The effects of cognitive decline in acute rehabilitation have been shown to persist after hospital discharge and may affect the psychological well-being of this clinical population. Factors such as education, smoking, drug use, and post-concussion symptoms may also influence cognitive impairment. Patients with SCI with anxiety show worsening cognitive functioning, leading to impaired working memory functioning and delayed memory functioning (41, 66). Among behavioral and psychiatric symptoms, some articles suggest that people with SCI may experience depression, and their risk of developing it, may increase over time. Anxiety symptoms can also be a precursor to depression, and these adjustment disorders can harm independence in many aspects of life, such as exercise, personal hygiene, and social interaction (67–69). Routine fatigue, pain, depression, and anxiety may be associated with poorer social participation. Despite these findings, depressive symptoms are mediated by appraisal and coping strategies, and general self-efficacy and high life purpose are among the protective factors (70, 71). In addition, symptoms of intense anxiety and depression are consistent predictors of various aspects of pain and stress (72, 73). When depression is present in these patients, suicidal ideation occurs as a psychiatric symptom, and resilience may be a protective factor against suicidal ideation. Finally, pain and fatigue are independently associated with depression in patients with SCI (74–76).

Scientific literature supports the idea that psychiatric and cognitive symptoms can affect the rehabilitation process. Cognitive impairment has a significant negative impact on functional outcomes after SCI, with little functional gain during rehabilitation (77). In addition, these individuals, with cognitive impairment, have been reported to have increased aggressive behaviors and a higher risk of rehospitalization (34). SCI patients may exhibit different patterns of cognitive performance depending on the degree of injury, and overall performance may differ significantly from healthy controls. Furthermore, these patients may have pre-illness difficulties that affect cognition (and increase the risk of SCI), such as learning disabilities, and substance abuse (78, 79). The literature highlights the fact that cognitive and psychiatric symptoms are linked, in fact, some consequences and complications of SCI, such as sleep-disordered breathing/sleep apnea, mental disorders, chronic pain, medication side effects, fatigue, and decreased physical activity, may affect cognition (80–84). For example, up to 50% of patients with high-grade SCI have sleep apnea and its severity may be directly related to cognitive impairment (85, 86). Several studies have shown that there is a significant negative correlation between the severity of depression and cognitive ability and that cognitive ability is a strong predictor of psychological impairment after hospital discharge (87). Looking at this issue from a rehabilitation perspective, four possible treatments have been explored to address cognitive impairment: 1) drug therapy (82, 84, 88), 2) percutaneous tibial nerve stimulation (89), 3) dietary therapy and supplements (90), and 4) inpatient rehabilitation (91, 92). Evidence on the effects of drug therapy dietary modification and supplement interventions on cognition in SCI is sparse and inconclusive (93), while percutaneous tibial nerve stimulation is a safe treatment for cognitive impairment after SCI (89). Combining several inpatient rehabilitation treatments has positive but heterogeneous effects on cognition.

From a psychiatric perspective, SCI patients experiencing psychological distress also show strong associations with measures of mood: both acceptability and self-efficacy are significantly negatively associated with depression, and the latter is also associated with anxiety, all of which may influence negative rehabilitation (94). The relationship between SCI and depressive symptoms is moderated by the quality of social support, the degree of conflict within the family, and the cognitive mediation of events. Major depressive episodes are more likely to occur when several psychosocial variables lead to prolonged feelings of hopelessness and helplessness. Frequent stressful events following an injury are likely to increase the patient’s feelings of helplessness and hopelessness. The impact of stressful events is therefore mediated by patients’ cognitive appraisals of their coping resources and their characteristic patterns of response to threatening events. Healthcare providers should assess the patient’s psychiatric and emotional problems and utilize the wide range of resources that may be available to the patient. Treatment professionals may need to assess the patient’s coping skills to anticipate adjustment problems. By facilitating access to patients’ adaptive personality traits, intellectual abilities, and social outlets, treatment can reduce the risk of psychiatric symptoms such as loss of interest, apathy, loneliness, loss of motivation, and depression (95). Cognitive therapy can also help reduce negative attitudes underlying depressed mood (96). To guarantee functional psychological rehabilitation in this patient population, the situation should be considered as a ‘non-isolated’ process involving the medical team, family, and close friends. All these people can play an important role in alleviating cognitive and psychological symptoms. Therefore, the goals of the rehabilitation team should include facilitating the family’s adaptation to changing circumstances (97). Furthermore, by understanding the influential variables associated with disability acceptance, rehabilitation educators and researchers can design professional training and research programs that fully consider the psychological, social, and occupational consequences of living with SCI (98).

This scoping review had several strengths. It is based on evidence from longitudinal observational populations and cross-sectional studies with large sample sizes. It includes an analysis of the cognitive impairments and psychiatric symptoms as well as some instruments to detect them and their disability. We have also identified data gaps in many areas, hopefully providing information for future research. This review has contributed to highlighting some cognitive, behavioral, and psychiatric aspects and symptoms of patients with SCI, often overshadowed compared to medical and biological problems from the point of view of the literature, through a selection of studies carried out over the last 10 years (from 2013 to 2023) and therefore based on recent data. Compared to past literature, this review allows us to infer that the psychological functioning (both cognitive and emotional) of the patient with SCI must be analyzed and monitored over time to avoid the evolution of a worse clinical picture characterized by various cognitive deficits (in information processing speed, working memory) or psychiatric symptoms (depression, anxiety, fatigue, stress). Particular attention must be paid to the living environment (together with age) and their behavioral patterns as these are two variables that can increase or decrease exposure to further risks (education, smoking, drug use, post-concussion symptoms) and connote different diagnosis and rehabilitation paths. Unlike previous literature, this study highlighted various potential environmental and subjective risk factors and identified functional coping mechanisms and resilience as protective factors for managing clinical symptoms, particularly psychiatric symptoms. Furthermore, its positive impact was also underscored.

The main limitation of the present study is the few papers that meet the inclusion criteria, as we included only fourteen articles that explored cognitive, behavioral, and psychiatric symptoms, and only four of them focused on the cognitive aspect. This, besides the heterogenous methodology and samples, prevents us from drawing robust evidence on this important topic. Four databases were also used, and the articles were restricted by date, so it is possible that important evidence was omitted. Furthermore, the sample size varies a lot: some are large, some are small, and the parameters measured are different. Clinicians in the rehabilitation field should recognize the potential for cognitive impairment, screen for such impairments, and provide proactive interventions for this population. Much information is needed to clarify the meaning of cognitive impairment to effectively improve cognitive impairment and maximize functional independence in the SCI population. Future research could also examine protective factors that reduce the likelihood of developing adjustment and mood disorders such as depression and anxiety after SCI, such as seeking social support rather than isolation and avoidance, and a tendency to engage in difficult emotional experiences.

In conclusion, this review shows that SCI patients may experience psychiatric symptoms and cognitive impairments that affect their functioning. At the same time, these patients may be more prone to various adjustment and mood disorders such as depression, stress, and anxiety. Moreover, these two aspects may interact with each other, causing a range of symptoms, increasing the risk of hospitalization, and delaying the rehabilitation process. Given the few studies included in our work, the conclusions that can currently be drawn are preliminary and the current evidence requires further investigations. Researchers should continue to study these clinical symptoms and disorders and their role in the rehabilitation process and develop practical interventions to be implemented as early as possible after a traumatic or non-traumatic SCI.

## Author contributions

AC: Data curation, Investigation, Methodology, Writing – original draft, Writing – review & editing. DC: Data curation, Formal analysis, Resources, Visualization, Writing – original draft, Writing – review & editing. RDL: Conceptualization, Formal analysis, Visualization, Writing – original draft, Writing – review & editing. AQ: Funding acquisition, Project administration, Supervision, Writing – original draft, Writing – review & editing. FC: Data curation, Visualization, Writing – original draft, Writing – review & editing. RC: Conceptualization, Methodology, Supervision, Writing – original draft, Writing – review & editing.
