# Supplementary material for: Cognitive, behavioral and psychiatric symptoms in patients with spinal cord injury: a scoping review
Source: Front Psychiatry. 2024 Mar 20;15:1369714. doi: 10.3389/fpsyt.2024.1369714 (PMC10987747; doi:10.3389/fpsyt.2024.1369714)
Supplement: Supplementary file 1 [file Table_1.docx]

**Table 1.** Summary of studies included in the research.

| **Author** | **Aim** | **Study Design/Intervention** | **Treatment Period** | **Sample Size** | **Outcomes Measures** | **Main Findings** | **Study Limitations** | **Statistical Analyses.** |
| --- | --- | --- | --- | --- | --- | --- | --- | --- |
| Cohen et al. 2017 [67] | To compare the cognitive profile of a well-characterized sample of adults with and without SCI using the NIHTBCB. | Multi-Site Study. | Not Specificated. | 312 Individuals (156 with SCI and 156 without it). | NIHTB-CB. | The NIHTB-CB is relatively fast, samples important cognitive domains, has good normative data, and is suitable for some people with SCI (those with functional use of one hand). People with SCI living in the community are at risk of mild cognitive impairment, especially in tasks that rely on processing speed and executive function. | Except for the NIHTB-CB episodic memory measure, testing was limited to examining processing speed and executive function. | Most analyses aimed to report the cognitive profile of SCI patients, so participants with incomplete data were excluded. Multivariate analysis of variance and analysis of covariance were used to compare SCI participants' subtest scores and composite scores with controls drawn from the NIHTB validation sample. |
| Javoroski et al. 2022 [68] | Examine the correlations with and without SCI. | Comparative Study. | Not Specificated. | 23 patients with SCI and 20 participants without SCI. | QALY, EQ-5D VAS, SCS-M, NIH Toolbox. | Dissociation between age, cognition, and QoL has occurred in SCI; the discrepancy between EQ-5D QALYs and VAS suggests that SCI patients may recalibrate their assessment of QoL to minimize the importance of mobility impairment. | Small sample size. The relatively high emotional health of the current SCI cohort may not be representative of the SCI population in general. | QoL differences between participants with SCI and without SCI were examined by Student’s t-test with Bonferroni adjustment for multiple comparisons (12 QoL scales: p = 0.00416). Cognitive differences were similarly examined (4 Toolbox indicators x 2 scale scores each: Bonferroni adjustment p = 0.00625). |
| Pasipanodya et al. 2020 [41] | To examine the determinants of cognitive function profiles in people undergoing acute inpatient SCI rehabilitation and the relationship between cognitive function and psychological well-being (life satisfaction and depression) six months after baseline assessment. | Prospective Observational Study Design. | Between September 2013 and October 2015. | 89 patients with SCI. | RBANS, LSI-A, PHQ-9. | The impact of cognitive impairment during acute rehabilitation may persist after hospital discharge and affect the psychological well-being of individuals with SCI. Identifying individuals with cognitive impairment and paying attention to modifiable risk factors can help improve post-SCI maladjustment. | The RBANS is a well-validated measure of cognitive function, but because it includes several motor-related subtests, it is not suitable for cognitive assessment of people with SCI with impaired upper motor function. | Baseline demographic, behavioral, medical, and cognitive differences between those who completed follow-up and those who lost follow-up were examined using t-tests, chi-square tests, and Mann-Whitney tests. |
| Carlozzi et al. 2021 [69] | Examines the relationship between physical and mental symptoms and cognition in people with SCI. | Observational Study. | 7 days of home monitoring, and a follow-up visit for each individual. | 174 Individuals with SCI. | HRQOL, PROMIS, CVLT-II, D-KEFS, PASAT. | There were generally no associations between symptom measures and neuropsychological test performance. However, anxiety and fatigue were associated with reduced self-cognitive functioning. | The SCI sample is relatively homogeneous (especially in terms of education) and the results may not generalize to the less educated segment in SCI. Although the data was collected in two different geographical regions, the sample was collected in a relatively affluent region. Approximately 1/3 of our sample is female which represents a slight overrepresentation of females in this sample relative to the broader SCI population. | A series of multivariate linear regressions were used to test the association between physical and mental symptoms and the seven cognitive function ratings, controlling for age, gender, education, and injury category. |
| Singh et al. 2022 [70] | This study examines depression to understand how autonomic regulation and coping after injury are related to physical and cognitive symptoms of depression three years later in patients with SCI. | Longitudinal Study Design. | Not Specificated. | 28 SCI participants. | PHQ-9, SCL-CSQ. | Depression scores decreased 3 years after injury (p≤0.05). The decline in depression scores at follow-up (T2) is consistent with a "disability paradox" and may indicate cognitive adaptation, especially for people who show autonomic adaptation in the form of high autonomic variability after injury. | Previous longitudinal studies have addressed the issue of attrition rates. The current results should be interpreted in the context of high attrition rates due to small sample sizes. | Coping and depression ratios were calculated and data analysis was performed using SPSS version 21 for Windows. Shapiro-Wilk test assessed the normality of PHQ-9 and SCL-CSQ scores. |
| Hara et al. 2022 [71] | To determine the predictive and multivariate associations of depression and anxiety symptoms with various sociodemographic and clinical variables in patients with SCI. | Cross Sectional Design. | From 2015 to 2019. | 556 patients. | HADS, FIM, MoCA. | No associations were found with alcohol or illicit drug use, level of injury, etiologic diagnosis, or duration of injury. For the anxiety model, the main predictor was depressive symptoms and injury-related characteristics were not significant; characteristics of the FIM scale and cognitive aspects of the MoCA scale were found to be the main predictors of depressive symptoms. Injury characteristics and movement disorders were not statistically significant. | The lack of normalized SCI population values for MoCA scale total score may affect this study. Lack of measures of pain and sleep. | Univariate and multivariate linear regression analyses were performed using anxiety and depression subdomains as dependent variables, demographic and clinical variables as independent variables, and hospital anxiety and depression scale scores. |
| Peter et al. 2015 [72] | Validate SCIAM and examine how psychological resources influence depressive symptoms in people with SCI. | Cross Sectional Survey. | Not Specificated. | 516 patients. | Self-Questionnaire, PIL-SF, ALE, HADS. | Psychological resources of SCI patients may have a direct impact on depressive symptoms; higher GSE and PIL are significantly associated with lower depressive symptoms. | Due to the cross-sectional study design, it is not possible to draw conclusions about causal relationships. To achieve an appropriate sample size/variable ratio, biological factors such as injury level and environmental factors such as social support were not included. Psychological factors can be represented by other variables such as optimism and extraversion. | Structural equation modeling was used to verify the relationships between the variables specified in SCIAM. |
| Ulrich et al. 2014 [73] | It examined the rates of psychiatric disorders associated with depression after SCI and the relationship between depression diagnosis and medical and medication use. | Retrospective Study. | Retrospective Analysis From 1997 to 2007. | 41.213 with SCI. | NARDEP, ICD-9. | People with SCI and depression are more likely to have co-occurring psychiatric disorders, resulting in increased healthcare visits and medication use. | The database was limited to investigating all research questions, such as definitions of comorbidities and health service utilization. | Descriptive statistics were used to examine questions about prevalence and sample characteristics. Chi-square analysis and t-tests were used to compare veterans diagnosed with a depressive disorder with veterans selected from the NARDEP random sample who were not diagnosed with depression. |
| Kuzu et al. 2022 [74] | To examine same-day associations between perceptions of pain, fatigue, depressed mood, anxiety, and cognitive function and social participation in daily life in adults with SCI. | Observational Study. | Between September 2017 and June 2019. | 168 individuals with SCI. | PROMIS, EOD diaries. | The results of the multivariate model showed that daily increases in fatigue (B = -0.10; P =.004) and depressive symptoms (B = -0.25; P =<.001) and cognitive decline (B = 0.11; P =<.001) were significantly associated with worse social engagement during the day. Diurnal variation in anxiety and pain was not associated with intraday social engagement. | Cross-sectional nature of the data; EOD has high measurement validity and reliability and causal interpretation of the effect of symptoms on patient functioning is not possible. It is not possible to causally interpret the effect of symptoms on patient functioning. | The data in this study are hierarchical and days are nested across individuals. A linear mixed-effects model was used to model the relationship between EOD symptom measures and social participation. |
| Murray et al. 2017 [75] | To determine the medical and psychological correlates of pain in people with childhood SCI. | Cross Sectional Study. | Not Specificated. | 187 adults with SCI. | BAI, PHQ. | The results revealed medical and psychological correlates of pain. Strong symptoms of depression and anxiety were strong and consistent predictors of various aspects of pain, beyond the effects of gender, injury-related characteristics, and secondary medical complications. | The results of this study may not be generalizable to patients with SCI from different centers or other ethnic groups. Data on pain medications lack specificity for inclusion in the analysis as it does not ask whether a particular medication is prescribed for pain, depression, or a combination of these conditions. | All analyses were performed using SPSS. To address the main aim of the study, a Pearson chi-square test was first performed to examine differences in levels of injury at the pain site. To examine the unique contribution of psychological functioning in predicting persistent pain outcomes, hierarchical multiple regression was performed, controlling for gender and medical characteristics in the first three stages. |
| Migliorini et al. 2013 [76] | To test whether people whose subjective well-being returns to the normal homeostatic range after SCI are more resilient and thus less at risk of emotional distress over time. | Longitudinal Study. | Not Specificated. | 21 Adults with chronic SCI. | COMQoL-A5, PWI, DASS-21. | Patients with chronic SCI may be vulnerable to mental health problems even if they have previously demonstrated good resilience, and their subjective well-being after SCI may not be as stable as the general QoL literature examining genetic and personality associations with subjective well-being suggests. | The small sample size available and the study's deliberate sampling methodology (i.e., sampling only those without evidence of mental distress at T1) may have influenced the study results. | Statistical analysis was performed using paired t-test for continuous data and Phi and Cramer's V χ2 test for discrete data; P values below 0.05 were considered statistically significant. |
| Saglam et al. 2023 [77] | To investigate depression, PTSD status, resilience, and SI and to examine predictors and clinical correlates of current SI in traumatic SCI. | Cross Sectional Study | Between May 2018 and May 2019. | 63 patients with SCI. | PCL-5, PHQ-9, BRS and Sociodemographic measures. | SI is highly prevalent in patients with traumatic SCI and is associated with psychiatric comorbidities such as depression and PTSD. Resilience (which is inversely related to SI and has protective and predictive value) is an important area for psychotherapeutic intervention and screening. | It is difficult to establish a causal relationship due to the cross-sectional design. Sample size, recruitment from a single center, and inclusion of SCI patients hospitalized for rehabilitation reduce the generalizability of the findings. | Statistical analysis was performed using SPSS. Patients were divided into two groups according to the presence or absence of SI. Kolmogorov-Smirnov test was used for normality test. Since continuous variables were not normally distributed, Mann-Whitney U test was used to compare the characteristics of the two groups. Independent sample t-tests were used to compare normally distributed variables. Levene's test was used to evaluate the equality of variance. |
| Alschuler et al. 2013 [78] | Describe the relationship between pain and fatigue and physical and psychological functioning in adults with SCI. | Cross Sectional Survey. | Not Specificated. | 540 Individuals. | PROMIS, PHQ-9, NMS. | Pain and fatigue were independently associated with depression, but only pain was associated with physical functioning. Furthermore, depression was more severe in middle-aged compared to younger and older age groups. Physical functioning decreased with increasing age and severity of injury. | It was a convenience sample of individuals who were willing to participate in the study. The cross-sectional nature of the study allows to identify only associations between variables without inferring causal relationships. Data were self-reported. | By calculating Pearson correlation coefficients between predictor and criterion variables and determining their zero-order correlations, the hypothesis that pain and fatigue are related to each other and physical functioning and depression was tested. |
| Min et al. 2014 [79] | To determine the moderating effect of resilience on the negative effects of chronic pain on depression and post traumatic growth. | Comparative Study. | From November 2010 to May 2011. | 37 Individuals with SCI. | CD-RISC, PHQ-9, PTGI. | Resilience had the potential to reduce the negative effects of pain. In addition, resilience contributed to reduced depression and post-traumatic growth. | The small sample size and single-center recruitment may contribute to selection bias and limit the generalizability of the findings; the low consent rate of 38.9% may be attributed to the short hospital stay and the difficulty of completing a paper-and-pencil formatted questionnaire due to quadriplegia. This may also contribute to selection bias. | After controlling for relevant covariates, hierarchical linear regression analyses were conducted to determine the moderating effects of resilience on the relationship between pain depression, and posttraumatic growth. |

**Legend: Spinal Cord Injury (SCI), NIH Toolbox – Cognition Battery (NIHTBCB), Quality of Life (QoL), Visual Analog Scale (VAS), Quality Adjusted Life-Year (QALY), Spinal Cord Injury Secondary Conditions Scale - Modified (SCS-M), National Institutes of Health Toolbox for Neurological and Behavioral Function - Cognition Battery (NIH Toolbox), Repeatable Battery for the Assessment of Neuropsychological Status (RBANS), Life Satisfaction Index A (LSI-A), Patient Health Questionnaire-9 (PHQ-9), Health-Related Quality of Life (HRQOL), Patient Reported Outcomes Measurement Information System (PROMIS), California Verbal Learning Test-II (CVLT-II), Delis-Kaplan Executive Function System (D-KEFS), Paced Auditory Serial Addition Test (PASAT), Spinal Cord Lesion Coping Strategies Questionnaire (SCL-CSQ), Hospital Anxiety and Depression Scale (HADS), Functional Independence Measure (FIM), Montreal Cognitive Assessment Scale (MoCA), Spinal Cord Injury Adjustment Model (SCIAM), Higher General Self-Efficacy (GSE), Higher Purpose in Life (PIL), Purpose in Life Test-Short Form (PIL-SF), Appraisal of Life Events Scale (ALE), National Registry for Depression (NARDEP), International Classification of Disease-9 (ICD-9), Patient-Reported Outcomes Measurement Information System (PROMIS), End-Of-Day (EOD), Beck Anxiety Inventory (BAI), Statistical Package for the Social Sciences (SPSS), Comprehensive Quality of Life Scale - Adult v5 (COMQoL-A5), Personal Well-being Index (PWI), Depression, Anxiety & Stress Scale - Short Form (DASS-21), Post-Traumatic Stress Disorder (PTSD), Suicidal Ideation (SI), PTSD Checklist for Diagnostic and Statistical Manual of Mental Disorders, Fifth Edition (PCL-5), Brief Resilience Scale (BRS), Numerical Rating Scale (NRS), Connor-Davidson Resilience Scale (CD-RISC), Posttraumatic Growth Inventory (PTGI).*
