# Supplementary material for: Cognitive, behavioral and psychiatric symptoms in patients with spinal cord injury: a scoping review
Source: Front Psychiatry. 2024 Mar 20;15:1369714. doi: 10.3389/fpsyt.2024.1369714 (PMC10987747; doi:10.3389/fpsyt.2024.1369714)
Supplement: Supplementary file 2 [file Table_2.docx]

Table 2. The description of tools for screening cognitive, disability functions, and psychiatric disorders following SCI.

| The Repeatable Battery for the Assessment of Neuropsychological Status (RBANS), is a simple tool that allows clinicians to rapidly assess a range of cognitive domains. It was developed for two purposes: identification and characterization of abnormal cognitive decline in the elderly and neuropsychological screening of younger patients. The whole battery takes less than 30 minutes. It consists of 12 subtests that contribute to total and index scores in five domains: immediate memory, visuospatial construction, language, attention, and delayed memory [40]. | |
| --- | --- |
| **Cognitive Domain** | **Description** |
| Immediate Memory | This index consists of the following two subtests: (a) List learning: involves the immediate recall of a 10-item word list over four learning trials. The words are semantically irrelevant, of the early age of acquisition, relatively rich in imagery, and as phonologically unique as possible. (b) Story Memory: a 12-item story is read over two trials. Recall is scored using verbatim criteria to avoid complex scoring rules [40]. |
| Visuospatial Construction | This index consists of the following two subtests: (a) Figure Copy: For the imitation of a graph, the patient copies a geometric shape consisting of 10 pieces. Each part is worth 2 points (accuracy and placement) for a total of 20 points. (b) Line Orientation: consists of a 10-item line orientation test. Each item contains a radial array of 13 lines at 180 degrees, with two target lines below the array in the same direction as the two lines in the array. The subjects' task on each item is to identify the matching lines. One point is awarded for each correctly matched line, for a total of 20 points [40]. |
| Language | This index consists of the following two subtests: (a) Picture Naming: Consists of 10-line drawings and the subject has to name the picture. If the object is misidentified, a semantic signal is provided (e.g., calling a mushroom "umbrella"). (b) Semantic fluency: includes the total number of examples produced within 60 seconds for a given semantic category (e.g., fruit or vegetable). The semantic categories used were chosen to try to minimize retrieval demands and therefore use semantic memory more specifically concerning retrieval strategies [40]. |
| Attention | This index consists of the following two subtests: (a) Digit Span: each input has two sequences of digits ranging in length from two to nine digits. The second sequence of a given length is read-only if the first sequence fails. (b) Coding: is similar to the Symbol Digit Modality Test and the WAIS-R Digit Symbol subtest. Numbers rather than symbols were chosen for responses to avoid the potentially detrimental effect of compositional misbehaviors on performance. Scoring was based on the total number of items completed within 90 seconds [40]. |
| Delayed Memory | This index consists of the following two subtests: (a) List recall: involves free recall of words from a list learning task. (b) List recognition: includes a yes/no recognition test on word memory of a list learning task. (c) Story recall: involves free recall of a story from a story memory test. (d) Recall of the Form: includes the free recall of a form from a Copy of the Form obedience test [40]. |
| The Primary and Secondary Disability Rating Scales (ADAPSS) assess 'the individual's initial assessment of an event or situation'. Secondary measures assess the individual's assessment of coping resources, the likelihood that these resources are adequate, and the likelihood that these resources can be used effectively. The scale consists of 6 subscales: 1) Fearful Despondency; 2) Overwhelming Disbelief; 3) Determined Resolve; 4) Growth and Resilience; 5) Negative Perceptions of Disability; 6) Personal Agency [42]. | |
| The Hospital Anxiety and Depression Scale (HADS) is widely used to estimate the prevalence of clinically significant anxiety and clinically significant depressive symptoms in adults in epidemiologic studies and to detect clinically significant symptoms in clinical settings The HADS was developed to detect clinically significant symptoms of anxiety and depression that do not include physical symptoms and consists of 14 items on a 1-4 Likert scale [43]. | |

**Legend: Repeatable Battery for the Assessment of Neuropsychological Status (RBANS), Wechsler Adult Intelligence Scale-Revised (WAIS-R), Primary and Secondary Disability Rating Scales (ADAPSS), Hospital Anxiety and Depression Scale (HADS).*
